# Functional Significance of Conflicting Age and Wealth Cross-Categorization: The Dominant Role of Categories That Violate Stereotypical Expectations

**DOI:** 10.3389/fpsyg.2016.01624

**Published:** 2016-10-21

**Authors:** Jingjing Song, Bin Zuo

**Affiliations:** School of Psychology, Central China Normal UniversityWuhan, China

**Keywords:** stereotype, cross-categorization, age, wealth, functional significance

## Abstract

The purpose of the current study was to identify the functional significance of conflicting stereotypes and to identify the dominant category in such conflicts. In the present research we examined the conflicting crossed categories of age and wealth with regard to warmth and competence perceptions. It was found (Pilot Study and Study 1) that the old-rich targets presented a conflicting stereotype group in the perception of warmth, whereas young-poor targets presented a conflicting stereotype group in the perception of competence. In addition, the *old* stereotype dominated the warmth evaluation of old-rich targets, whereas the *poor* stereotype dominated the competence evaluation of young-poor targets. In Study 2, participants provided warmth and competence evaluations after they learned about the targets' behaviors which demonstrated high or low warmth and high or low competence. The results suggest that for the warmth evaluation of the old-rich target the category that did not match the behavior (i.e., contradicted the stereotype expectation) was more salient and drove judgments. However, the effect of stereotype expectation violation was not found in the competence evaluation of the young-poor target. The results are discussed in terms of their implications for understanding factors that activate and inhibit stereotyped perceptions.

## Introduction

People live in complicated societies and may belong to many social categories simultaneously. Importantly, how a person is perceived may vary depending on those categories or combinations of categories. Cross-categorization refers to the process of classifying persons according to two categories. Recently, research has begun to explore people's evaluations of persons belonging to crossed-categories (Urada et al., 2007; Bodenhausen, 2010; Sesko and Biernat, 2010; Freeman and Ambady, 2011; Johnson et al., 2012; Neuberg and Sng, 2013; Penner and Saperstein, 2013; Kang and Bodenhausen, 2015). The accessibility and functional significance of each category can affect perceptions of the cross-categorized target. The functional significance of a category refers to the dominance of the category's influence on the perceiver's evaluations of a target in a specific situation It has been demonstrated that the perceiver's attributes and the context can influence the functional significance of a category when considered in isolation. Extending previous research (Turner et al., 1987; Crisp and Hewstone, 2006), in the present study we focused on the age and wealth crossed-category and tested the co-effect of the perceiver's attributes (attitudes toward each simple-category) and the context (the behavior of the target) on functional significance.

### The conflicting natural and social crossed categories

Previous research on the perception of cross-categorized targets focused on explicit and relatively unmodifiable natural categories, such as gender or race, including the cross of race and gender (Johnson et al., 2012; Klauer et al., 2014; Schug et al., 2015), race and age (Kang et al., 2014), and age and gender (Klauer et al., 2003; Cloutier et al., 2014). One exception was a study by Smith et al. (1996), in which both categories were social categories (i.e., a target could be categorized as a baseball player and a gambler as well).

Major natural categories dominate the early stages of person perception (Dovidio et al., 1997; Ma and Correll, 2011). However, when the perceiver has sufficient time, capacity, and motivation, targets will be categorized in terms of multiple categories (Pendry and Macrae, 1996), which include natural categories as well as social categories. In this context social categories may play an important role in stereotype evaluation. One social category that perceivers commonly use is Social Economic Status, and based on the wealth component of this category, individuals can be classified as rich and poor targets. This category was one focus of the current study.

Stereotypes about the rich and poor can be conceptualized in terms of Fiske et al. (2002) stereotype content model (SCM), which assumes that people tend to evaluate others based on the two dimensions of warmth and competence. Competence refers to position in the status power hierarchy, whereas warmth refers to cooperation within one's own group (Abele and Wojciszke, 2014). The stereotype link to the rich is low warmth and high competence (Piff et al., 2010). However, the poor target is perceived as having low warmth and low competence (Fiske et al., 2002). The negative stereotype of the poor may decrease their probability of receiving an equal professional development opportunity, and perceptions of an imbalance in social wealth distribution may lead to hatred of the rich, thus further increasing the risk of social instability. Therefore, research on the wealth category is of great applied value.

With regard to the natural category examined in this study, we focused on age, which has received less attention than other major natural categories such as sex or race. The old target is perceived as showing high warmth (Kite et al., 2005; Chasteen et al., 2012) and low competence (Hess et al., 2009; Eich et al., 2014), whereas the young target is perceived as showing low warmth and high competence (Song et al., in press).

The old-rich target elicits conflicting stereotypes, as there is a high warmth and low competence evaluation for *old*, but a low warmth and high competence evaluation for *rich*. The young-poor target is also in a conflicting stereotype group as there is a high competence evaluation of the young target and low competence evaluation of the poor target. In cross-categorization involving conflicting stereotypes, the two sub-categories, which refer to *old* and *rich* categories when evaluating the old-rich target, and *young* and *poor* categories when evaluating the young-poor target, are subject to a competition for mental dominance, and they may not have equal psychological significance to the perceiver (Crisp and Hewstone, 2006). Thus, the salient and dominant category in conflicting crossed-categories (i.e., the category with high functional significance) can determine the perception of this target as negative or positive. In the current study, we only focused on this aspect of cross-categorized groups, namely the functional significance of conflicting stereotypes.

### The relation between the perceiver's attributes and functional significance

In the case of cross-categorization, the most relevant and accessible category will stand out (Bodenhausen, 2010), and when context information is not given, the degree of accessibility is determined by two factors about the perceiver's attributes. First, accessibility is determined by the strength of the perceiver's attitude (stereotype) toward the relevant categories. The category about which people have strong attitudes tends to attract attention and to be the dominant category (Fazio et al., 1995; Crisp and Hewstone, 2006). Second, accessibility is determined by the perceiver's past experience in categorizing a particular person or other social object. The perceiver who had previously judged the target as belonging to one category would likely categorize the target in a similar way in the future (Smith et al., 1992).

SCM predicts conflicting stereotypes of old-rich targets with respect to both warmth and competence, but for young-poor targets, the conflicting stereotype occurs only with respect to competence. Thus, we focused on the functional significance of the categories *old* and *rich* with respect to evaluations of both warmth and competence, but for the categories of *young* and *poor* we focused only on the evaluation of competence. The stereotype strength is the key factor influencing the dominant category (Fazio et al., 1995; Crisp and Hewstone, 2006). However, the warmth stereotype strength is similar for old and rich targets (Fiske et al., 2002). Thus, it is difficult to recognize the dominant category of the old-rich target in the warmth evaluation only based on the stereotype strength. Furthermore, as a fundamental natural category, age is more visible and easily identifiable than wealth, and thus it is more accessible. Therefore, age is repeatedly used to categorize people in daily life, and this repeated practice may make the age category more accessible to the perceiver than the wealth category. Thus, we assumed that in the warmth evaluation of the conflicting categorization (old-rich), the age category would be the dominant category (H1).

For the competence evaluation of the old-rich and young-poor targets, the age category is also more accessible. However, the perception of competence is always closely connected with a person's wealth (Cuddy et al., 2009), and the stereotypes of competence in relation to the wealth category were shown to be stronger than the stereotypes of competence in relation to the age category in a previous investigation (Fiske et al., 2002). Thus, the competence evaluation results in an emphasis on the wealth category. We assumed that in the competence evaluation of conflicting categories (old-rich and young-poor), the wealth category would be the dominant category (H2).

### The relation between context information and functional significance

Context information and specific mental schemas (stereotypes) that are triggered by a combination of categories can influence perception of the crossed-category target (Casper et al., 2011). The parallel constraint satisfaction model suggests that different context information activates different subsets of a network of connections, and thus the dominant category and the evaluation of the target would change across contexts (Kunda et al., 1997; Crisp and Hewstone, 2006). One important question is how context moderates perceptions of crossed-category targets, to understand what circumstances give rise to activation of some stereotype components and inhibition of others.

One form of context can be situational factors, e.g., a white female target in a group of black and white men (Van Rijswijk and Ellemers, 2002), and it has been demonstrated that the category that is unique, clear, and prominent in the situation is the dominant category. Context can also be the behavior of the target, and it was this kind of context that was examined in the current study on the age and wealth cross-categorization. Specifically, we analyzed the co-effect of (a) the perceiver's attitude (stereotype) toward the relevant categories and (b) context in the form of the target's specific behaviors, on the functional significance of each category.

Turner et al. (1987) showed that, when the behavior is consistent with the stereotype of a particular category (nominative fit), this category would be the dominant category, as the behavior information directs the perceiver to the stereotype-consistent category. However, a large number of studies since then have found that the perceiver pays attention to the target that contradicts the stereotype expectancy (Bettencourt et al., 1997; Dickter and Gyurovski, 2012; Garcia-Marques et al., 2016; Jerónimo et al., 2016). People engage in more effortful cognitive processing (Jerónimo et al., 2016), reorganize the “wrong description” (contradicting the expectancy), perceive the stereotype-inconsistent target as atypical, make more explanations about and prefer external attributions for the behavior (Sekaquaptewa et al., 2003; Sekaquaptewa and Espinoza, 2004), and make the stereotype-inconsistent behavior conform to their stereotype. Furthermore, the shifting standards model suggests that the target is evaluated with reference to the stereotype expectations of that particular category; the perceiver makes extreme judgments (Biernat and Vescio, 2002) and uses ironic language more often in response to the target who behaves in a manner contrary to expectation (Burgers and Beukeboom, 2014), so as to maintain stereotypic expectancies. Thus, the category associated with counter-stereotypic behavior attracts more attention, and we suspected that these categories may dominate the perception of cross-categorized targets.

In the warmth evaluation of the conflicting categorization of old-rich targets, we assumed that counter-stereotypic behavior would dominate the perception of cross-categorized targets. When the old-rich target shows low warmth behavior, the low warmth behavior contradicts the stereotype expectation of someone who is old. Thus, the *old* category would be expected to be the dominant categorization in the perception of the old-rich target that shows low warmth behavior (H3a). By contrast, the *rich* categorization would be the dominant categorization in the perception of the old-rich target that shows high warmth behavior (H3b). Furthermore, in the competence evaluation of the conflicting cross-categorized target (i.e., young-poor and old-rich), when the target fails, the *young* categorization should be the dominant categorization for the young-poor target, and the *rich* categorization should be the dominant categorization for the old-rich target (H3c); when the target succeeds, the *poor* categorization should be the dominant categorization for the young-poor target, and the *old* categorization should be the dominant categorization for the old-rich target (H3d).

### Overview of the current study

We conducted a pilot study and two larger studies to test the functional significance of conflicting stereotypes, focusing on the cross-categorization of age and wealth. The purpose of the pilot study was to verify that these categories did generate conflicting stereotypes, and furthermore to evaluate the functional significance of each category in cross-categorized groups. A categorization task was used in which the participants categorized the target (using both simple categories and crossed categories) as showing high competence or low competence and as showing high warmth or low warmth. In Study 1, the participants used rating scales to evaluate the warmth and competence of the young, the old, the rich, the poor, the old-rich and the young-poor target. Regression analysis was conducted to test the effect of attitude about the simple-category target on the perception of the cross-categorized target, and relative weight analysis was conducted to quantitatively analyze the functional significance of each category. Study 2 tested the functional significance in specific scenarios to determine whether the dominant and weaker categories varied depending on context, and tested the co-effect of the “attitude about the simple-category” and the “behavior of the target” on the perception of the cross-categorization. The dependent measures included direct (warmth and competence evaluations) and indirect (attributions) stereotype evaluations.

## Pilot study: the examination of age and wealth categories in terms of the SCM and qualitative analysis of functional significance

The first purpose of the pilot study was to verify the SCM in relation to the age and wealth categories. We expected to find that *old* would be the high warmth and low competence group, *young* and *rich* would be the high competence and low warmth groups, and *poor* would be the low warmth and low competence group, constituting conflicting stereotype groups. The second purpose of the pilot study was to qualitatively explore the functional significance of the conflicting stereotypes.

### Method

#### Participants

A total of 45 students from a university in central China volunteered to participate in this study. Data from one additional participant were not included in the analysis because of an incomplete categorization task. The participants' ages ranged from 18 to 30 years (*M* = 22.78, *SD* = 3.00), and there were 18 males (40.0%) and 27 females (60.0%).

#### Materials and procedure

Permission was obtained from the university ethics committees. Participants were recruited and invited to the laboratory where they were introduced to the categorization task. After receiving a description of the study, participants gave written informed consent. They received a small gift (candy) at the end of the study.

The Categorization Task: The task materials consisted of eight “identity cards.” Each identity card presented a very simple head-and-shoulders photo silhouette in black-and-white on the left side of the card (Dommelen et al., 2015) and the identity information in text on the right side of the card. Samples of identity cards can be seen in the Supplementary Material. The participants could not recognize the gender or age through the photos, and the identity text was the only useful information. On four identity cards, a simple category was depicted (*old, young, poor*, and *rich*). Crossing wealth and age led to four category conjunctions, which were presented on the other four cards (the old-rich, the young-rich, the old-poor, and the young-poor). Each identity card was presented three times to each participant.

The first task was the warmth categorization, in which participants placed targets into one of two boxes labeled “high warmth (kindness and friendliness)” and “low warmth (kindness and friendliness).” The second task was the competence categorization, in which participants placed targets into one of two boxes labeled “high competence (confidence and intelligence)” and “low competence (confidence and intelligence).” After being shown an example identity card, participants were asked to categorize the full set of cards into the two boxes (high vs. low warmth and high vs. low competence). The cards were presented in random order, and the participants were given enough time to evaluate the targets and finish the task. Participants were de-briefed after the task.

The frequency with which each participant assigned a specific card into the high competence box or the high warmth box was calculated, and it ranged from 0 to 3 because each card was presented three times. The categorization task has been widely used in previous research by assigning the target to the “in-group (us)” box or the “out-group (not us)” box, and it has been demonstrated to be an effective task to categorize the cross-categorized target (Singh et al., 1997; Dommelen et al., 2015).

### Results

Chi-square tests were conducted to compare the number of people choosing the high competence/warmth box for each target 0, 1, 2, or 3 times with the expected value of 45/4 = 11.25. As shown in Table 1, the results showed that 23 subjects assigned all three *old* targets into the high warmth box, which is significantly higher than the expected value (χ^2^ = 21.76, *p* < 0.001). The frequencies with which participants placed all three young targets into the high competence (*n* = 29) and high warmth boxes (*n* = 20) were also significantly higher than the expected value (χ^2^ = 47.36, *p* < 0.001; χ^2^ = 10.20, *p* < 0.05). As for wealth, the frequencies with which subjects placed all three rich targets into the high competence (*n* = 34) and high warmth (*n* = 3) boxes and assigned all three poor targets into the low competence (*n* = 31) box were both significantly different compared with the expected value of 11.25 (χ^2^ = 65.13, *p* < 0.001; χ^2^ = 12. 51, *p* < 0.01; χ^2^ = 50.20, *p* < 0.001). The results indicate that the young-poor target presents a conflicting stereotype group in the competence evaluation, and the old-rich target presents a conflicting stereotype group only in the warmth evaluation.

**Table 1:** **Absolute frequency of categorizations for the high warmth and the high competence category in the pilot study**.

	**High warmth category**				**High competence category**			
	**0**	**1**	**2**	**3**	**0**	**1**	**2**	**3**
Old	2	7	13	23	12	13	8	12
Rich	14	19	9	3	1	1	9	34
Young	6	8	11	20	1	1	14	29
Poor	7	10	18	10	31	10	3	1
Old-rich	3	8	12	22	1	3	6	35
Young-poor	12	10	17	6	21	5	11	8
Old-poor	7	13	11	14	38	5	1	1
Young-rich	16	9	14	6	5	3	7	30

N = 45, the data in the table refers to the number of subjects assigned targets 0, 1, 2, or 3 times into the high warmth/competence category. As young-poor target presents a conflicting stereotype group in the competence evaluation (young target is perceived as high competence, and poor target is perceived as low competence), and great majority of subjects assigned all three young-poor targets into the low competence category. Thus, we could assume the strength of the high competence stereotype of the young target was lower than the strength of the low competence stereotype of the poor target, and the poor was the primary category in the competence evaluation of the young-poor target.

Furthermore, the frequencies with which participants placed all three old-rich targets into the high warmth (*n* = 22) box and high competence (*n* = 35) box, and assigned all three young-poor targets into the low competence (*n* = 21) box were significantly higher than the expected value of 11.25 (χ^2^ = 17.31, *p* < 0.01; χ^2^ = 67.98, *p* < 0.001; χ^2^ = 12.87, *p* < 0.01). Thus, for warmth evaluations of the old-rich target, *old* was the primary category, and in the competence evaluation of the young-poor target, *poor* was the primary category. H1 was supported. H2 originally made predictions about both the competence evaluation of the young-poor target and of the old-rich target, but we were only able to test the former component because in our pilot study *old* and *rich* did not constitute a conflicting cross-category in the competence evaluation. Thus, the dominance of the wealth category could not be fully confirmed. The results regarding the young-rich and old-poor combinations can be seen in Appendix A (Supplementary Material).

### Discussion of pilot study

The results of the pilot study do not fully comply with the SCM, as old targets were not perceived as incompetent. One possible methodological reason is that the term “old” used in the stimulus material may have been interpreted differently by the participants than was intended, and there may be large differences especially concerning competence if one thinks of an “old” person who is 55 or 90 years old. Another reason may be that part of the definition of competence, namely intelligence, could have been interpreted as meaning either fluid intelligence or crystallized intelligence, or both. Furthermore, young targets were perceived as warm rather than cold. This may be because the participants were in-group members of the *young* category, and thus they have made positive evaluations of the young target.

Although not fully consistent with the SCM, the pilot data do provide two examples of conflicting stereotype groups (old-rich target in the warmth evaluation and young-poor target in the competence evaluation), and these could be used as stimuli in Studies 1 and 2. Furthermore, the results showed that, *old* was the dominant category in the warmth evaluations of the old-rich target, and *poor* was the dominant category in the competence evaluation of the young-poor target.

There were limitations in the identity card categorization task used in the pilot study. Participants could only classify targets as being in the “high” or “low” warmth/competence-group, and there was no “middle” or “cannot decide” category. This may lead to an overestimation of stereotypical trait ascriptions, as subjects were forced to choose either the “high” or the “low” box even if they had no clear preference. In addition, using each identity card three times to represent the distinct categories and category conjunctions artificially increases power. Furthermore, demand characteristics may have played a role, and some participants might have been aware of what was being measured by the task.

The pilot study identified the dominant category in the warmth evaluation of the old-rich target, and in the competence evaluation of the young-poor target. However, the pilot study did not assess the relative weight of each category quantitatively. Thus, quantitative analyses were needed to directly compare the relative weight of each category.

## Study 1: the relative weight of simple categories in the perception of conflicting stereotypes

The pilot study demonstrated the functional significance of each of the simple categories to which the old-rich and young-poor targets belonged. However, the pilot study did not assess stereotype quantitatively, and it did not calculate the relative weight of each category in the stereotype evaluations of the cross-categorized targets. In Study 1, we used regression analysis to directly examine the relative importance (weight) of each category by asking the participants to use rating scales to evaluate the warmth and competence of targets belonging to simple categories and crossed-categories.

### Methods

#### Participants

A total of 104 students from a university in central China participated in this study. The participants' ages ranged from 17 to 23 years (*M* = 19.38, *SD* = 1.17), and there were 20 males (19.2%) and 84 females (80.8%). There were 51 participants from rural areas (49.0%) and 53 from the city (51.0%). When asked to rate how wealthy they were, three participants described themselves as “very poor” (2.9%), 29 participants described themselves as “rather poor” (27.9%), 62 participants described themselves as “average” (59.6%), and 10 participants described themselves as “rather rich” (9.6%).

#### Materials and procedure

Permission was obtained from the university ethics committees to conduct this study. The questionnaire was administered to the students in a class during one class period. Two trained data collectors administered the questionnaire according to a manual of procedures to standardize the data collection process. Participants gave written informed consent after receiving a description of the study, and they received a small gift (candy) at the end of the study.

The participants were asked to evaluate the warmth and competence of six targets (the old, the rich, and the old-rich targets; the young, the poor, and the young-poor targets). As an introduction, participants were told that this was a social perception task, and they were asked to evaluate some strangers' personalities on the basis of a limited amount of information. The six identity targets were presented on identity cards (refer to pilot study). The presentation order of the targets was counterbalanced, and it complied with the principle that the first two cards presented were simple-category cards (e.g., old, rich), followed by a card crossing the two simple categories (e.g., old-rich). Competence was evaluated with three traits: competence, intelligence, and confidence. Warmth was also evaluated with three traits: warmth, friendliness, and kindness (Fiske et al., 2002; Judd et al., 2005). Participants were asked to rate each adjective according to its descriptiveness of the target on a scale from 1 (*not at all descriptive*) to 5 (*very descriptive*). The sum of the three items (warmth or competence) was the final score. The higher the score was, the higher the perceived competence or warmth of the target was. The participants were then de-briefed. This method, measuring explicit attitudes toward the target, has been widely used in previous research, and it has been shown to be valid (Judd et al., 2005; Corcoran et al., 2009; Kang et al., 2014). In the current study, the internal consistency reliability (α) was 0.76 for the competence measure and 0.86 for the warmth measure.

### Results

The correlations presented in Table 2 indicated that the warmth evaluation of the old-rich target was significantly positively correlated with the warmth evaluation of the rich target and of the old target. Additionally, the competence evaluation of the young-poor target was significantly positively correlated with the competence evaluation of the poor target, although not correlated with the competence evaluation of the young target. The results about the correlation among the competence ratings for old, rich, and old-rich target, and the warmth ratings for young, poor, and young-poor targets can be seen in Appendix B (Supplementary Material).

**Table 2:** **Correlations among all the variables in study 1 and descriptive information**.

	**1. Old warmth**	**2. Rich warmth**	**3. Old-rich warmth**	**4. Young competence**	**5. Poor competence**	**6. Young-poor competence**
1	−					
2	0.28^**^	−				
3	0.60^**^	0.49^**^	−			
4	0.31^**^	0.32^**^	0.37^**^	−		
5	0.17	0.23^*^	0.15	0.22^*^	−	
6	0.02	−0.05	0.20^*^	0.12	0.35^**^	−
*M*	10.79	8.00	10.45	10.85	8.34	8.71
*SD*	2.15	2.20	2.27	1.82	2.17	2.20

N = 104,

* p < 0.05,

** p < 0.01, scale range: 3–15.

We next used linear regressions to explore the relationship between the evaluation of targets belonging to the simple categories and crossed categories. For the warmth evaluation of the old-rich target, we conducted a regression analysis to test the warmth evaluations of the old and rich targets as predictors of the warmth evaluation of the old-rich target. The first block included the demographic variables: age, gender, wealth, and Hukou (a family registration program that serves as a domestic passport and divides residents into two groups: urban and rural). The second block included the warmth evaluation of the rich and the old targets. The warmth evaluation of the old-rich target was the dependent variable. As can be seen in Table 3, the regression analysis indicated that, after controlling for the demographic variables, the warmth evaluations of the old target and the rich target were significantly associated with the warmth evaluation of the old-rich target (β = 0.51, *p* < 0.001; β = 0.34, *p* < 0.001). Next we tested if one of the simple categories was dominant. Relative weight (*RW*) analysis is a useful technique to calculate the relative importance of predictors (independent variables) when they are correlated with each other (LeBreton and Tonidandel, 2008). This analysis indicated that the relative weight of the *old* category (*RW* = 0.30) was greater than the relative weight of the *rich* category (*RW* = 0.17), providing further support for H1.

**Table 3:** **Hierarchical Linear Models of simple-category evaluations in relation to crossed-category evaluations (*N* = 104)**.

	**Warmth evaluation**				**Competence evaluation**		
	**β**	***t***	***RW***		**β**	***t***	***RW***
Age	0.10	0.12		Age	0.10	0.93	
Gender	0.03	0.32		Gender	−0.05	−0.50	
Wealth	0.07	0.87		Wealth	0.02	0.15	
Hukou	−0.03	−0.38		Hukou	−0.17	−1.76	
Old	0.51	6.44^***^	0.30 (62.84%)	Young	0.04	0.42	0.01 (6.65%)
Rich	0.34	4.39^***^	0.17 (37.16%)	Poor	0.35	3.56^**^	0.11 (93.35%)
*R*^2^ = 0.48				*R*^2^ = 0.17			

RW = raw relative weight, and numbers in brackets refer to rescaled relative weight estimates reported as percentage of predicted variance. Hukou is a household registration system in China, and it includes two types: rural and city, 1 = city, 2 = rural. For Gender, 1 = male, 2 = female;

** p < 0.01,

*** p < 0.001.

In the competence evaluation of the young-poor target, we also conducted a regression to analyze the competence evaluations of the young and poor targets as predictors of the competence evaluation of the young-poor target. As can be seen in Table 3, the results indicated that, after controlling for the demographic variables, the competence evaluation of the poor target was a significant predictor of the competence evaluation of the young-poor target (β = 0.35, *p* < 0.01), but the competence evaluation of the young target was not a significant predictor (β = 0.04, *p* > 0.05). The relative weight of the *poor* category (*RW* = 0.11) was greater than the relative weight of the *young* category (*RW* = 0.01), providing further support for H2 in the competence evaluation of the young-poor target. However, the competence evaluation of old-rich target was not tested in the current paper as the old-rich target was not a conflicting cross-category in the competence evaluation, and so the dominance of the wealth category in the competence evaluation of the old-rich target could not be confirmed. The results on the competence ratings of the old, rich, and old-rich, and warmth ratings of the young, poor, and young-poor can be seen in Appendix C (Supplementary Material).

### Discussion of study 1

The results of Study 1 were consistent with the results of the pilot study, further documenting that in the warmth evaluation of the old-rich target, the *old* category was the dominant category, and in the competence evaluation of the young-poor target, the *poor* category was the dominant category. Moreover, the results of Study 1 indicated that the stereotype evaluation of the simple category was positively related to the stereotype evaluation of the crossed category, and the strength of the perceiver's stereotype of each category determined the dominant category in the perception of the crossed-category target. In addition, as the context plays an important role in the functional significance of conflicting stereotypes, in Study 2 we tested the functional significance in specific scenarios.

## Study 2: the scenario specificity of functional significance

Study 2 tested the functional significance of conflicting stereotypes in specific scenarios. Participants were asked to use rating-scales to provide a direct index of their evaluations of the warmth or competence of targets with different behaviors. Moreover, inspired by the stereotype explanatory bias approach (Sekaquaptewa et al., 2003; Sekaquaptewa and Espinoza, 2004), the participants were asked to make attributions about the behavior of the target, and the attributions were taken as an indirect index of their perceptions of warmth or competence.

### Methods

#### Participants

A total of 156 students from a university in central China participated in this study. Of these participants, 95 evaluated the warmth of the old, the rich, and the old-rich targets, and 61 evaluated the competence of the young, the poor, and the young-poor targets. The participants' ages ranged from 17 to 27 years (*M* = 19.81, *SD* = 1.66), and there were 41 males (26.3%) and 115 females (73.7%). There were 69 participants from rural areas (44.2%) and 87 from the city (55.8%). When asked to rate how wealthy they were, 5 participants described themselves as “very poor” (3.2%), 36 participants described themselves as “rather poor” (23.1%), 110 participants described themselves as “average” (70.5%), and 5 participants described themselves as “rather rich” (3.2%).

#### Materials and procedure

Permission was obtained from the university ethics committees to conduct this study. Participants volunteered to participate for extra course credit. They provided informed consent and were de-briefed after the study. They received a small gift (candy) for their participation.

Six psychology doctoral students screened and chose four scenarios (high competence, low competence, high warmth, and low warmth) from the Judd et al. (2005) list of scenarios, and the scenario nominated most in each category was the final scenario used for that category. The following scenarios were chosen: one high warmth scenario (*X helped a blind woman cross the street*), one low warmth scenario (*X could not be bothered to give directions to a stranger*), one high competence scenario (*X won the yearly award for the employee who contributes most to the company's profits*), and one low competence scenario (*X failed a job interview*).

The introduction was the same as in Study 1. The description of the stranger added information about context in the form of behavior information. In order to minimize the demand characteristics and prevent the participants from guessing the purpose of the study, participants were randomly assigned to finish one of two tasks: (a) evaluate the high or low warmth behavior of the old, rich, and old-rich targets, or (b) evaluate the high and low competence behavior of the young, poor, and young-poor targets. The presentation order of the two scenarios (high and low competence or warmth) was random. For each scenario, the participants were firstly asked to evaluate the warmth or competence of two simple-category targets (e.g., *X was an old or rich person, and X helped a blind woman cross the street*), and then asked to evaluate the warmth or competence of the crossed-category target (e.g., *X was an old-rich person, and X helped a blind woman cross the street*). The warmth and competence rating scales were the same as in Study 1. In the current study, warmth and competence evaluation measures both had good reliability (α = 0.86, α = 0.78).

Moreover, after the direct warmth or competence evaluation, the participants were told to think carefully about why the stranger was engaging in the behavior based on the limited amount of information provided, and they were asked to write down one plausible explanation. The participants were being asked to make an attribution about the high or low competence behavior of the young, poor, and young-poor targets or about the high or low warmth behavior of the old, rich, and old-rich targets. Participants were de-briefed after the study.

#### Coding of attributions

In the current study, each attribution was rated by the research team on a five-point scale based on the attribution positivity. A positive attribution means that in the participant's view, the target is showing high warmth or high competence. Coding was conducted in three steps: (1) creation of an attribution table, (2) creation of a coding manual, and (3) conversion. In the first step, two experts in the stereotype field reviewed all attributions provided by all the participants, and these attributions were classified based on shared semantic meaning. They discussed any disagreements and compiled an attribution table based on consensus. The categories of the attributions in each scenario can be seen in Appendix D (Supplementary Material).

In the second step, creation of a coding manual, six doctoral students in the stereotype field rated the positivity of each category of attributions summarized in the first step. Five points were used, ranging from 1 (*in the participant's view, X was not at all warm or competent*) to 5 (*in the participant's view, X was very warm or competent*). The higher the score was, the higher the attribution positivity was. The integer of the average of the six raters was the final “attribution positivity” score for that type of attribution. For example, in the warmth evaluation of the old-rich target who engaged in high warmth behavior, the semantic category “the target helped a blind woman cross the street because of external benefit (he/she wanted to get a tip)” suggests that the participant made an external attribution rather than an internal attribution for the targets' helpful behavior; that is, the participant viewed the target as showing low warmth, and the attribution would be given a low score (1) on the attribution positivity scale. By contrast, the semantic category “the target helped a blind woman cross the street because of personal internal attributes (he/she is a very warm and friendly person)” suggests that participant attributed the target's helpful behavior to internal rather than external causes; that is, the participant viewed the target as showing high warmth, and the attribution would receive a high score (5) on the attribution positivity scale. These ratings were used as guides for specific scoring of each participant's attributions.

In the third step, two other postgraduates who majored in psychology and were blind to the hypotheses of the study converted all of the participants' handwritten attributions into numeric values (1–5) according to the coding manual made in step two. The sum of the two raters' scores was the final score of the attribution positivity, and we calculated Kendall coefficients to demonstrate the inter-rater reliability. For the low warmth behavior, the Kendall coefficients were 0.95 (rich), 0.91 (old), and 0.95 (old-rich). For the high warmth behavior, the Kendall coefficients were 0.86 (rich), 0.81 (old), and 0.80 (old-rich). For the low competence behavior, the Kendall coefficients were 0.81 (poor), 0.84 (young), and 0.79 (young-poor). For the high competence behavior, the Kendall coefficients were 0.86 (poor), 0.86 (young), and 0.74 (young-poor). This method of rating attributions has been demonstrated as valid (Song et al., in press), and the attribution positivity was used as an indirect index of stereotype evaluation.

### Results

#### Correlation analysis

The correlation analysis results can be seen in Tables 4, 5. The results indicated that the warmth evaluation of the old-rich target was significantly correlated with the warmth evaluation of the rich and old targets in both high and low warmth scenarios. The young-poor target competence evaluation was also significantly correlated with the young and poor target competence evaluations in both high and low competence scenarios.

**Table 4:** **Correlations among warmth evaluations and attributions about the behavior of the old, rich, and old-rich targets *(N* = 95)**.

	**Warmth evaluation**						**Attribution**					
	**Low warmth behavior**			**High warmth behavior**			**Low warmth behavior**			**High warmth behavior**		
	**1. Old**	**2. Rich**	**3. Old rich**	**4. Old**	**5. Rich**	**6. Old rich**	**7. Old**	**8. Rich**	**9. Old rich**	**10. Old**	**11. Rich**	**12. Old rich**
1	–											
2	0.46^**^	–										
3	0.61^**^	0.48^**^	–									
4	0.04	−0.002	0.06	–								
5	0.04	0.08	0.12	0.44^**^	–							
6	−0.02	0.03	0.20	0.38^**^	0.51^**^	–						
7	0.42^**^	0.18	0.18	−0.13	−0.02	−0.07	–					
8	0.22^*^	0.48^**^	0.23^*^	−0.01	0.22^*^	−0.04	0.23^*^	–				
9	0.15	0.28^**^	0.54^**^	−0.05	−0.01	0.04	0.33^**^	0.10	–			
10	−0.14	−0.09	−0.11	0.19	0.01	0.09	−0.02	−0.06	−0.07	–		
11	−0.04	−0.01	0.07	0.13	0.40^**^	0.20^*^	−0.15	0.07	−0.11	0.15	–	
12	0.01	0.06	0.06	−0.14	0.01	0.18	−0.10	−0.12	−0.05	0.12	0.28^**^	–
*M*	8.54	6.61	7.36	13.01	12.12	12.56	8.37	5.06	6.57	9.37	8.76	9.25
*SD*	2.25	2.22	2.89	1.67	1.98	2.23	1.99	2.89	3.00	0.97	2.34	1.64

* p < 0.05,

** p < 0.01.

**Table 5:** **Correlations among competence evaluations and attributions about the behavior of the young, poor, and young-poor targets *(N* = 61)**.

	**Competence evaluation**						**Attribution**					
	**Low competence**			**High competence**			**Low competence**			**High competence**		
	**1. Young**	**2. Poor**	**3. Young poor**	**4. Young**	**5. Poor**	**6. Young poor**	**7. Young**	**8. Poor**	**9. Young poor**	**10. Young**	**11. Poor**	**12. Young poor**
1	–											
2	0.36^**^	–										
3	0.35^**^	077^**^	–									
4	0.03	0.30^*^	0.27^*^	–								
5	−0.15	0.22	0.08	0.50^**^	–							
6	0.03	0.08	0.08	0.61^**^	0.71^**^	–						
7	0.07	−0.18	−0.24	0.01	0.11	0.12	–					
8	−0.07	0.20	0.18	0.10	0.08	−0.01	0.01	–				
9	−0.17	0.20	0.41^**^	0.05	0.10	0.07	0.29^*^	0.39^*^	–			
10	0.22	−0.02	0.10	−0.12	0.07	−0.02	−0.24	−0.06	−0.08	–		
11	0.03	0.15	0.23	0.21	0.19	0.29^*^	−0.07	0.19	0.19	0.09	–	
12	0.16	0.16	0.27^*^	−0.08	−0.13	−0.01	−0.10	−0.21	0.09	0.24	0.34^*^	–
*M*	9.38	9.00	8.34	12.49	12.30	12.77	6.72	5.57	5.95	9.56	8.62	9.13
*SD*	1.59	1.83	2.04	1.51	1.49	1.64	1.91	1.58	1.74	0.96	0.90	0.92

* p < 0.05,

** p < 0.01.

In the low warmth scenario, attributions for the old-rich target's behavior were positively correlated with attributions for the old target's behavior. In the high warmth scenario, however, attributions for the old-rich target's behavior were positively correlated with attributions for the rich target's behavior. Moreover, attributions for the young-poor target's behavior were significantly correlated with those for the young and poor targets' behavior in the low competence scenario, but only significantly correlated with those for the poor target's behavior in the high competence scenario.

#### The moderating role of scenario in the relations among warmth evaluations of the old, rich, and old-rich targets

Hierarchical linear models were conducted to explore the moderating role of the scenario in the relations among the warmth evaluations of the old, rich, and old-rich targets. The first block included the warmth evaluation of the old target, the warmth evaluation of the rich target, and the scenario. Scenario was a dummy variable, with the low warmth or low competence scenario assigned 0, and the high warmth or high competence scenario assigned 1. The second block included two interaction terms, which were computed as the product of scenario and the mean-centered measure of the warmth evaluation of the old or rich target. The third block included the product term of the three independent variables. As shown in Table 6, in the second model, the product term of scenario and old target warmth evaluation was significant (β = −0.17, *p* < 0.05). To further examine this two-way interaction, follow-up regressions were conducted for both the high and low warmth scenarios.

**Table 6:** **Hierarchical Linear Models of the moderating role of scenario in the relation between the simple-category stereotype evaluations and crossed-category stereotype evaluation**.

		**Warmth or competence evaluation**		**Attribution**	
		**β**	***t***	**β**	***t***
Model 1	Old	0.40	5.76^***^	0.24	3.66^***^
	Rich	0.37	4.87^***^	0.11	1.42
	Scenario	0.11	1.57	0.35	4.65^***^
		*R*^2^ = 0.68		*R*^2^ = 0.31	
Model 2	Old	0.51	5.88^**^	0.28	3.88^***^
	Rich	0.32	3.08^**^	0.04	0.37
	Scenario	0.14	1.86^†^	0.36	4.63^***^
	Scenario × old	−0.17	−2.09^*^	−0.09	−1.22
	Scenario × rich	0.08	0.89	0.11	1.15
		*R*^2^ = 0.69		*R*^2^ = 0.32	
Model 3	Old	0.51	5.87^***^	0.28	3.89^***^
	Rich	0.32	3.07^**^	0.04	0.37
	Scenario	0.13	1.54	0.37	4.74^***^
	Scenario × old	−0.16	−1.59	−0.15	−1.74^†^
	Scenario × rich	0.08	0.65	0.06	0.61
	Scenario × rich × old	−0.01	−0.07	0.12	1.38
		*R*^2^ = 0.69		*R*^2^ = 0.32	
Model 1	Young	0.16	2.75^**^	0.24	3.27^**^
	Poor	0.60	10.13^***^	0.38	4.60^***^
	Scenario	0.23	3.85^***^	0.30	3.07^**^
		*R*^2^ = 0.83		*R*^2^ = 0.66	
Model 2	Young	0.09	1.08	0.25	3.06^**^
	Poor	0.67	8.99^***^	0.41	4.22^***^
	Scenario	0.24	3.96^***^	0.33	2.98^**^
	Scenario × young	0.12	1.59	−0.03	−0.32
	Scenario × poor	−0.11	−1.50	−0.05	−0.48
		*R*^2^ = 0.83		*R*^2^ = 0.67	
Model 3	Young	0.09	1.11	0.25	3.05^**^
	Poor	0.67	9.19^***^	0.41	4.20^***^
	Scenario	0.20	3.43^**^	0.28	1.96^†^
	Scenario × young	0.24	2.76^**^	0.03	0.19
	Scenario × poor	−0.03	−0.42	0.01	0.05
	Scenario × young × poor	−0.19	−2.50^*^	−0.07	−0.44
		*R*^2^ = 0.84		*R*^2^ = 0.67	

Scenario was a dummy variable, with the low warmth or competence scenario was 0, high warmth or competence scenario was 1. Interaction terms were computed as the product of scenario and the mean-centered measure of the warmth/competence evaluation of the simple category target(s).

† p < 0.1,

* p < 0.05,

** p < 0.01,

*** p < 0.001.

As can be seen in Table 7, in the low warmth scenario, the results of the linear regression indicated that, after controlling for the demographic variables, the *old* warmth evaluation and the *rich* warmth evaluation accounted for significant variance in the old-rich warmth evaluation (β = 0.49, *p* < 0.001; β = 0.26, *p* < 0.01), and the relative weight of the *old* category (*RW* = 0.28) was greater than that of the *rich* category (*RW* = 0.14). In regard to the high warmth scenario, the warmth evaluations of the *old* and the *rich* targets both significantly predicted the old-rich warmth evaluation (β = 0.22, *p* < 0.05; β = 0.44, *p* < 0.001), and the relative weight of the *rich* category (*RW* = 0.20) was greater than that of the *old* category (*RW* = 0.09). H3a and H3b were supported. The category with stereotype-inconsistent behavior was the dominant category.

**Table 7:** **Hierarchical Linear Models of evaluations of simple-category targets in relation to evaluations of crossed-category targets in specific scenarios**.

**Dependent variables**	**Independent variables**	**Low warmth or competence**			**High warmth or competence**		
		**β**	***t***	***RW***	**β**	***t***	***RW***
Warmth evaluation of the old-rich target	Age	0.03	0.37		0.11	1.11	
	Gender	−0.06	−0.65		0.09	0.95	
	Wealth	−0.11	−1.21		0.27	2.83^**^	
	Hukou	0.04	0.49		−0.07	−0.74	
	Old	0.49	5.43^***^	0.28 (66.18%)	0.22	2.24^*^	0.09 (29.93%)
	Rich	0.26	2.83^**^	0.14 (33.82%)	0.44	4.49^***^	0.20 (70.07%)
		*R*^2^ = 0.44			*R*^2^ = 0.36		
Attributions about the old-rich target	Age	−0.10	−0.91		−0.33	−0.30	
	Gender	−0.00	−0.02		−0.13	−1.20	
	Wealth	−0.16	−1.45		−0.08	−0.74	
	Hukou	−0.08	−0.70		0.24	2.10^*^	
	Old	0.33	3.12^**^	0.10 (94.53%)	0.09	0.88	0.01 (12.92%)
	Rich	−0.00	−0.02	0.01 (5.47%)	0.18	1.71^†^	0.07 (87.08%)
		*R*^2^ = 0.15			*R*^2^ = 0.14		
Competence evaluation of the young-poor target	Age	0.31	3.56^**^		0.19	1.97^†^	
	Gender	0.03	0.32		0.07	0.70	
	Wealth	−0.06	−0.67		−0.03	−0.30	
	Hukou	0.13	1.40		−0.07	−0.71	
	Young	0.04	0.43	0.06 (10.86%)	0.30	3.11^**^	0.23 (38.67%)
	Poor	0.77	8.57^***^	0.53 (89.14%)	0.52	5.09^***^	0.36 (61.33%)
		*R*^2^ = 0. 68			*R*^2^ = 0. 62		

RW = raw relative weights, and numbers in brackets refer to rescaled relative weight estimates reported as percentage of predicted variance. Hukou is a household registration system in China, and it includes two types: rural and city.

† p < 0.1,

* p < 0.05,

** p < 0.01,

*** p < 0.001.

#### The moderating role of scenario in the relations among attribution positivity ratings of the old, rich, and old-rich targets

In order to explore the moderator role of the scenario in the relations between the attribution positivity ratings of the rich and old targets and the attribution positivity ratings of the old-rich targets, hierarchical linear models were conducted. As can be seen Table 6, the results showed that the product term of the scenario and the attribution for the old target's behavior was marginally significant (β = −0.15, *p* = 0.08). We conducted two follow-up regressions for the high and low warmth behaviors to further explore this two-way interaction.

As shown in Table 7, in the low warmth scenario, the regression analysis indicated that, after controlling for the demographic variables, attributions for the old target's behavior significantly positively predicted attributions for the old-rich target's behavior (β = 0.33, *p* < 0.01); however, attributions for the rich target's behavior did not predict attributions for the old-rich target's behavior (β = −0.002, *p* > 0.05). Thus, the relative weight of the *old* category (*RW* = 0.10) was greater than that of the *rich* category (*RW* = 0.01). In the high warmth scenario, attributions for the rich target's behavior marginally positively predicted attributions for the old-rich target's behavior (β = 0.18, *p* < 0.1); however, attributions for the old target's behavior did not predict attributions for the old-rich target's behavior (β = 0.09, *p* > 0.05). Thus the relative weight of the *rich* category (*RW* = 0.07) was greater than that of the *old* category (*RW* = 0.01). H3a and H3b were also supported.

#### The moderating role of scenario in the relations among competence evaluations of the young, poor, and young poor-targets

In order to analyze how the scenario moderated the relations between the competence evaluations of the young and poor targets and the competence evaluation of the young-poor target, hierarchical linear models were used. As can be seen in Table 6, the results of this third model indicated that the product term of the three variables was significant (β = −0.19, *p* < 0.05). Follow-up linear regressions were conducted for both the high and low competence scenarios to further examine this three-way interaction.

As can be seen in Table 7, for the low competence scenario, the results of the linear regression indicated that, after controlling for the demographic variables, the competence evaluation of the poor target was a significant predictor of the young-poor competence evaluation (β = 0.77, *p* < 0.001), but the competence evaluation of the young target was not a significant predictor (β = 0.04, *p* > 0.05). Thus, the *poor* category had a greater relative weight (*RW* = 0.53). For the high competence scenario, the regression analysis showed that the competence evaluations of the young and the poor targets were both significantly positively associated with the young-poor competence evaluation (β = 0.30, *p* < 0.01; β = 0.52, *p* < 0.001). The relative weight of the *poor* category (*RW* = 0.36) was greater than that of the *young* category (*RW* = 0.23). H3c was not supported, but H3d was supported in the competence evaluations of the young-poor target. However, the competence evaluation of old-rich target was not tested, and so the dominance of the category that contradicted the stereotype expectation in the competence evaluation of the old-rich target could not be confirmed.

#### The moderating role of scenario in the relations among attribution positivity ratings of the young, poor, and young-poor targets

Hierarchical linear models were conducted to explore the moderator effect of scenario in the relation between the attribution positivity ratings of the young and poor targets and the attribution positivity ratings of the young-poor target. As shown in Table 6, the product term was not significant in either Model 2 or Model 3. The Model 1 indicated that attributions for the *poor* target's behavior and attributions for *young* target's behavior could predict attribution for the young-poor target' behavior (β = 0.41, *p* < 0.001; β = 0.25, *p* < 0.01). The relative weight analysis indicated that the *poor* category (*RW* = 0.38) was relatively more important than *young* category (*RW* = 0.25). H3c was not supported, but H3d was supported in the attributions made for the young-poor target's behavior. However, attributions for the high or low competence of the old-rich target were not tested, and so the dominance of the category that contradicted the stereotype expectation in the indirect competence evaluation of the old-rich target could not be confirmed.

### Discussion of study 2

There was some evidence that the scenario (high vs. low warmth behavior) moderated the functional significance of the young and poor categories in the explicit competence evaluation. In the high competence scenario, *poor* was the dominant category. In the low competence scenario, the relative weight of the *poor* category was less than in the high competence scenario, but was still dominant. However, the moderator effect was not supported by the indirect attribution measurement. This may be because the attribution measure, as an indirect indicator of attitude, is not sensitive enough to detect the moderator effect. With regard to the functional significance of the *old* and *rich* categories, the scenario specificity of the results was verified both in the direct warmth or competence evaluations and in the indirect attribution positivity scores. The two methods obtained relatively consistent results: The *old* category was the dominant category in the perception of the old-rich target's low warmth behavior, whereas the *rich* category was the dominant category in the perception of the old-rich target's high warmth behavior. There were also some additional findings in the regression model. Specifically, for the warmth evaluation of the old-rich target, the warmth evaluation of the old and rich were both significant predictors. In contrast, for the attribution measure, only the attribution for the old target's behavior could predict the attribution for the old-rich target's behavior in the low warmth scenario, and only the attribution for the rich target's behavior was a significant predictor in the high warmth scenario. We suspect that the perceiver would be likely to evaluate the target based on the two given categories, and the weight of the two categories would determine perception of the crossed-category in the explicit evaluation. However, in the implicit evaluation, the perceiver may only perceive the target based on one dominant category, and the cognition process in the implicit evaluation would be likely to take the shortcut because it is simpler and more concise.

There were some limitations in Study 2. First, only one low warmth scenario, one high warmth scenario, one low competence scenario, and one high competence scenario were used. Although a rigorous process was performed for choosing the appropriate scenarios, the suitability and feasibility of these scenarios still need to be established. Moreover, the rigorous procedure of selecting scenario settings reduces the ecological validity and generalization of the conclusions. Second, it is still necessary to explore whether the causal attribution (external/internal) could be mixed up with trait ascription. For example, an external attribution for high warmth behavior might reflect less trait ascription (in this case “warm”) than an internal attribution for high warmth behavior. The analysis of the attribution positivity requires careful consideration in specific contexts.

## General discussion

The purpose of the current study was to test the functional significance of conflicting stereotypes (i.e., old-rich and young-poor), and to identify the dominant category and the weaker category in these cross-categorizations. The pilot study used a categorization task to verify that these were conflicting categories by identifying perceptions of these categories in relation to warmth and competence. In Study 1, the participants were asked to use rating scales to evaluate the competence and warmth of targets belonging to simple and crossed categories. The results in both the pilot study and Study 1 showed that the *old* category was the dominant category in the warmth evaluation of the old-rich target, and the *poor* category was the dominant category in the competence evaluation of the young-poor target. This shows that the stereotype related to the simple-category was positively associated with the stereotype related to the crossed-category, and the category with the stronger stereotype was the dominant category in the perception of the crossed-category. Study 2 further tested the functional significance of these categories in specific scenarios, and the results varied depending on the situation-dependent behavior of the target. An old-rich target who behaves warmly is judged more in line with one's evaluations regarding the rich, whereas an old-rich target that behaves un-warmly is judged more in line with one's evaluations regarding the old. However, in the competence evaluation of the young-poor target, *poor* was the dominant category in both high and low competence scenarios. Thus, the hypothesis that the category that contradicts the stereotype expectation is potentially more salient and drives judgments is partly supported.

The *rich* category was the dominant category for an old-rich target that behaves warmly, whereas the *old* category was the dominant category for an old-rich target that behaves un-warmly. The inconsistent conclusions demonstrate the scenario-specificity in the functional significance analysis of old-rich groups (Casper et al., 2011, 2015). As was evident in our findings, it is the stereotype related to a certain category combined with behavior of the target that affects the functional significance (Crisp and Hewstone, 2006), and violations of stereotypic expectancy (the high warmth behavior of the rich target, and the low warmth behavior of the old target) attract more attention (Bettencourt et al., 1997; Dickter and Gyurovski, 2012). It may be because the category that violates the stereotype expectancy would be salient and relatively more accessible, and thus, would be selected as the dominant category. But further research is still needed to determine exactly how this process unfolds.

In the competence evaluation of the young-poor target, we found *poor* was the primary category when the behavior information was not given. Consistent with our hypotheses, the category with the stronger stereotype was the dominant category, and the low competence stereotype of the poor target was much stronger than the high competence stereotype of the young target. When considering the information about the target's behavior, we obtained consistent results with *poor* always being the dominant category. The dominance of the stereotype-inconsistent category in evaluation of cross-categorized targets was shown in the warmth-evaluation of the old-rich targets, but not found in the competence-evaluation of the young-poor targets. This might be due to the content of the dependent variable (warmth or competence) as well as the stereotype content (stereotype evaluation of the rich, old, young, and poor targets). We suspect that when there was a stronger stereotype of a certain category, any moderator effect of the additional behavior information would be lessened. The *poor* category dominated the competence ratings relatively independent of scenario. Regardless of the young-poor target's behavior, the perceiver would be likely to evaluate the target based on the *poor* category.

There is also another possibility. Most of the participants in Studies 1 and 2 saw themselves as “average” (neither poor nor rich), and thus the *poor* as well as the *rich* category constituted the out-group for most. However, most participants were young and may have seen themselves as in-group members of the *young* category. The results showing that additional behavioral information affected the evaluation of the old-rich target, but not the young-poor target, may have occurred because that stereotype-inconsistent information only dominates the evaluation of cross-categorized targets if the stereotype relates to an out-group category, but not to an in-group category. Further research is needed to verify this assumption.

It should be noted that participants may know examples of particular subtypes of persons, for example philanthropic old, rich people who are warm and caring, and young college students who came from a poor family, but have ambition and ability. Therefore, subtypes, rather than superordinate categories, may be driving participants' decisions. In addition, when additional identities like gender and race are not specified, participants may impose “male” and “in-group” identities on to the targets they are imagining (Cuddy et al., 2015). There also is the possibility that perceptions may be specific to old-rich men or old-rich women based on the previous experience of the perceiver, and the old-rich men may be evaluated differently from old-rich women in terms of stereotype-based assumptions about how targets acquired their wealth. Perceivers would make a positive competence evaluation of the old-rich men if they make the stereotypic assumption that old-rich men earn the wealth themselves, but may make a negative competence evaluation of old-rich women if they assume they acquired the wealth through a relationship with a rich partner.

Although we acknowledge that we have not conclusively pinpointed the mechanism underlying the functional significance of conflicting stereotypes, our studies do suggest some clues. We explored the co-effect of the stereotype related to a certain category and the behavior of the target. The results indicated that, in the warmth evaluation of the old-rich target, the category that showed behavior contrary to the stereotype expectation was the dominant category, but this was not found in the competence evaluation of the young-poor target. These findings are partly consistent with previous research showing that the target whose behavior violates the stereotype expectation attracts more attention, but we have extended this research by demonstrating the effect of stereotype expectation violation on the functional significance of the conflicting old and rich categories in the warmth evaluation. In addition, we extended research on the effect of stereotype expectation violation to examine context as a moderator of this effect. Here, there is no theoretical explanation for why context in the form of target behaviors moderated the salience of some categories but not others, although we speculate that this effect may disappear in the in-group evaluation, or be reduced when there is a very strong stereotype related to warmth or competence of the category. From a more practical and applied point of view, knowledge of functional significance obtained from the current study can be utilized to help individuals to find more effective intervention strategies designed to reduce prejudice. It will be important in future research to determine whether intervention targeting the dominant category or the “other” category will be most useful for reducing stereotypes.

We also make a methodical contribution. The pilot study used a categorization task to identify the functional significance of the conflicting stereotypes, and Study 2 extended the stereotypic explanatory bias approach to study perceptions of cross-categorization groups based on participants' attributions. Sekaquaptewa et al. (2003) posited that subtracting the number of explanations (internal or external) for stereotype-consistent events from the number of explanations provided for stereotype-inconsistent events provides an indirect measure of the stereotype. In contrast, in the current study we coded the attribution based on the attribution positivity, which is a more sensitive index compared with the type of attribution (internal or external attribution).

With regard to other limitations and potential extensions of the current work there are several issues worthy of note. First, members of an “out-group” based on one category may be evaluated more positively if they are also members of an “in-group” based on another category. The sample in the current study was made up of young, educated, mostly female participants, most of whom probably identify themselves as members of the *young* category. As this may influence the results, further research should include older participants, so as to take into consideration the role of the intergroup identity. Future research on this issue is important, as in-group identification is one important mechanism for reducing prejudice and defamation against a cross-categorized group (Crisp et al., 2003; Ray et al., 2010). Second, further study should also focus on sub-categories like middle-class (rather than rich and poor) and middle-aged (as opposed to old and young) targets. Moreover, other categories such as gender and non-dichotomous categories such as race (African American, Asian, White, etc.) need more attention. Third, the current research only focused on conflicting cross-categorization, but an analysis of the functional significance of consistent cross-categorization might also prove valuable, and future research on this topic is needed.

## Ethics statement

All procedures performed in studies involving human participants were in accordance with the ethical standards of the institutional and/or national research committee, the American Psychological Association (APA) standards and with the 1964 Helsinki declaration and its later amendments or comparable ethical standards.

## Author contributions

JS conceived and designed the experiments, analyzed and interpreted the data, wrote the report. BZ had role in the study design.

### Conflict of interest statement

The authors declare that the research was conducted in the absence of any commercial or financial relationships that could be construed as a potential conflict of interest. The reviewer PS and the handling Editor declared their shared affiliation, and the handling Editor states that the process nevertheless met the standards of a fair and objective review.

## References

[B1] Abele A. E. Wojciszke B. (2014). Communal and agentic content in social cognition: a dual perspective model. Adv. Exp. Soc. Psychol. 50, 195–255. 10.1016/B978-0-12-800284-1.00004-7

[B2] Bettencourt B. A. Dill K. E. Greathouse S. A. Charlton K. Mulholland A. (1997). Evaluations of ingroup and outgroup members: the role of category-based expectancy violation. J. Exp. Soc. Psychol. 33, 244–275. 10.1006/jesp.1996.1323

[B3] Biernat M. Vescio T. K. (2002). She swings, she hits, she's great, she's benched: implications of gender-based shifting standards for judgment and behavior. Pers. Soc. Psychol. Bull. 28, 66–77. 10.1177/0146167202281006

[B4] Bodenhausen G. V. (2010). Diversity in the person, diversity in the group: challenges of identity complexity for social perception and social interaction. Eur. J. Soc. Psychol. 40, 1–16. 10.1002/ejsp.647

[B5] Burgers C. Beukeboom C. J. (2014). Stereotype transmission and maintenance through interpersonal communication the irony bias. Commun. Res. 43, 414–441. 10.1177/0093650214534975

[B6] Casper C. Rothermund K. Wentura D. (2011). The activation of specific facets of age stereotypes depends on individuating information. Soc. Cogn. 29, 393–414. 10.1521/soco.2011.29.4.393

[B7] Casper C. Rothermund K. Wentura D. (2015). Automatic stereotype activation is context dependent. Soc. Psychol. 41, 131–136. 10.1027/1864-9335/a000019

[B8] Chasteen A. Kang S. Remedios J. (2012). Aging and stereotype threat: development, process, and interventions, in Stereotype Threat: Theory, Process, and Application, Vol. 2, eds Inzlicht M. Schmader T. (New York, NY: Oxford University Press), 202–216.

[B9] Cloutier J. Freeman J. B. Ambady N. (2014). Investigating the early stages of person perception: the asymmetry of social categorization by sex vs. age. PLoS ONE 9:e84677. 10.1371/journal.pone.0084677 24465423 PMC3897390

[B10] Corcoran K. Hundhammer T. Mussweiler T. (2009). A tool for thought! When comparative thinking reduces stereotyping effects. J. Exp. Soc. Psychol. 45, 1008–1011. 10.1016/j.jesp.2009.04.015

[B11] Crisp R. J. Hewstone M. (2006). Multiple Social Categorization: Context, Process, and Social Consequences. Hove; New York, NY: Psychology Press.

[B12] Crisp R. J. Hewstone M. Richards Z. Paolini S. (2003). Inclusiveness and crossed categorization: effects on co-joined category evaluations of in-group and out-group primes. Br. J. Soc. Psychol. 42, 25–38. 10.1348/014466603763276108 12713754

[B13] Cuddy A. J. Fiske S. T. Kwan V. S. Glick P. Demoulin S. Leyens J. P.. (2009). Stereotype content model across cultures: towards universal similarities and some differences. Br. J. Soc. Psychol. 48, 1–33. 10.1348/014466608X314935 19178758 PMC3912751

[B14] Cuddy A. J. Wolf E. B. Glick P. Crotty S. Chong J. Norton M. I. (2015). Men as cultural ideals: cultural values moderate gender stereotype content. J. Pers. Soc. Psychol. 109, 622–635. 10.1037/pspi0000027 26414843

[B15] Dickter C. Gyurovski I. (2012). The effects of expectancy violations on early attention to race in an impression-formation paradigm. Soc. Neurosci. 7, 240–251. 10.1080/17470919.2011.609906 21919565

[B16] Dommelen A. Schmid K. Hewstone M. Gonsalkorale K. Brewer M. (2015). Construing multiple ingroups: assessing social identity inclusiveness and structure in ethnic and religious minority group members. Eur. J. Soc. Psychol. 45, 386–399. 10.1002/ejsp.2095

[B17] Dovidio J. F. Kawakami K. Johnson C. Johnson B. Howard A. (1997). On the nature of prejudice: automatic and controlled processes. J. Exp. Soc. Psychol. 33, 510–540. 10.1006/jesp.1997.1331

[B18] Eich T. S. Murayama K. Castel A. D. Knowlton B. J. (2014). The dynamic effects of age-related stereotype threat on explicit and implicit memory performance in older adults. Soc. Cogn. 32, 559–570. 10.1521/soco.2014.32.6.559

[B19] Fazio R. H. Jackson J. R. Dunton B. C. Williams C. J. (1995). Variability in automatic activation as an unobtrusive measure of racial attitudes: a bona fide pipeline? J. Pers. Soc. Psychol. 69, 1013–1027. 10.1037/0022-3514.69.6.1013 8531054

[B20] Fiske S. T. Cuddy A. J. Glick P. Xu J. (2002). A model of (often mixed) stereotype content: competence and warmth respectively follow from perceived status and competition. J. Pers. Soc. Psychol. 82, 878–896. 10.1037/0022-3514.82.6.878 12051578

[B21] Freeman J. B. Ambady N. (2011). A dynamic interactive theory of person construal. Psychol. Rev. 118, 247–249. 10.1037/a0022327 21355661

[B22] Garcia-Marques T. Mackie D. M. Maitner A. T. Claypool H. M. (2016). Moderation of the familiarity-stereotyping effect: the role of stereotype fit. Soc. Cogn. 34, 81–96. 10.1521/soco.2016.34.2.81

[B23] Hess T. M. Hinson J. T. Hodges E. A. (2009). Moderators of and mechanisms underlying stereotype threat effects on older adults' memory performance. Exp. Aging Res. 35, 153–177. 10.1080/03610730802716413 19280445 PMC2664156

[B24] Jerónimo R. Volpert H. I. Bartholow B. D. (2016). Event-related potentials reveal early attention bias for negative, unexpected behavior.Soc. Neurosci. [Epub ahead of print]. 10.1080/17470919.2016.1144646 PMC5567733 26821691

[B25] Johnson K. L. Freeman J. B. Pauker K. (2012). Race is gendered: how covarying phenotypes and stereotypes bias sex categorization. J. Pers. Soc. Psychol. 102, 116–131. 10.1037/a0025335 21875229

[B26] Judd C. M. James-Hawkins L. Yzerbyt V. Kashima Y. (2005). Fundamental dimensions of social judgment: understanding the relations between judgments of competence and warmth. J. Pers. Soc. Psychol. 89, 899–913. 10.1037/0022-3514.89.6.899 16393023

[B27] Kang S. K. Bodenhausen G. V. (2015). Multiple identities in social perception and interaction: challenges and opportunities. Annu. Rev. Psychol. 66, 547–574. 10.1146/annurev-psych-010814-015025 25061671

[B28] Kang S. K. Chasteen A. L. Cadieux J. Cary L. A. Syeda M. (2014). Comparing young and older adults' perceptions of conflicting stereotypes and multiply-categorizable individuals. Psychol. Aging 29, 469–481. 10.1037/a0037551 25244468

[B29] Kite M. E. Stockdale G. D. Whitley B. E. Johnson B. T. (2005). Attitudes toward younger and older adults: an updated meta-analytic review. J. Soc. Issues 61, 241–266. 10.1111/j.1540-4560.2005.00404.x

[B30] Klauer K. C. Ehrenberg K. Wegener I. (2003). Crossed categorization and stereotyping: structural analyses, effect patterns, and dissociative effects of context relevance. J. Exp. Soc. Psychol. 39, 332–354. 10.1016/S0022-1031(03)00017-9

[B31] Klauer K. C. Hölzenbein F. Calanchini J. Sherman J. W. (2014). How malleable is categorization by race? Evidence for competitive category use in social categorization. J. Pers. Soc. Psychol. 107, 21–40. 10.1037/a0036609 24956312

[B32] Kunda Z. Sinclair L. Griffin D. (1997). Equal ratings but separate meanings: stereotypes and the construal of traits. J. Pers. Soc. Psychol. 72, 720–734. 10.1037/0022-3514.72.4.720

[B33] LeBreton J. M. Tonidandel S. (2008). Multivariate relative importance: extending relative weight analysis to multivariate criterion spaces. J. Appl. Psychol. 93, 329–345. 10.1037/0021-9010.93.2.329 18361636

[B34] Ma D. S. Correll J. (2011). Target prototypicality moderates racial bias in the decision to shoot. J. Exp. Soc. Psychol. 47, 391–396. 10.1016/j.jesp.2010.11.002

[B35] Neuberg S. L. Sng O. (2013). A life history theory of social perception: stereotyping at the intersections of age, sex, ecology (and race). Soc. Cogn. 31, 696–711. 10.1521/soco.2013.31.6.696

[B36] Pendry L. F. Macrae C. N. (1996). What the disinterested perceiver overlooks: goal-directed social categorization. Pers. Soc. Psychol. Bull. 22, 249–256. 10.1177/0146167296223003

[B37] Penner A. M. Saperstein A. (2013). Engendering racial perceptions an intersectional analysis of how social status shapes race. Gender Soc. 27, 319–344. 10.1177/0891243213480262

[B38] Piff P. K. Kraus M. W. Côté S. Cheng B. H. Keltner D. (2010). Having less, giving more: the influence of social class on prosocial behavior. J. Pers. Soc. Psychol. 99, 771–784. 10.1037/a0020092 20649364

[B39] Ray D. G. Way N. Hamilton D. L. (2010). Crossed-categorization, evaluation, and face recognition. J. Exp. Soc. Psychol. 46, 449–452. 10.1016/j.jesp.2009.12.001

[B40] Schug J. Alt N. P. Klauer K. C. (2015). Gendered race prototypes: evidence for the non-prototypicality of Asian men and Black women. J. Exp. Soc. Psychol. 56, 121–125. 10.1016/j.jesp.2014.09.012

[B41] Sekaquaptewa D. Espinoza P. (2004). Biased processing of stereotype-incongruency is greater for low than high status groups. J. Exp. Soc. Psychol. 40, 128–135. 10.1016/S0022-1031(03)00093-3

[B42] Sekaquaptewa D. Espinoza P. Thompson M. Vargas P. von Hippel W. (2003). Stereotypic explanatory bias: implicit stereotyping as a predictor of discrimination. J. Exp. Soc. Psychol. 39, 75–82. 10.1016/S0022-1031(02)00512-7

[B43] Sesko A. K. Biernat M. (2010). Prototypes of race and gender: the invisibility of Black women. J. Exp. Soc. Psychol. 46, 356–360. 10.1016/j.jesp.2009.10.016

[B44] Singh R. Yeoh B. S. Lim D. I. Lim K. K. (1997). Cross-categorization effects in intergroup discrimination: adding versus averaging. Br. J. Soc. Psychol. 36, 121–138. 10.1111/j.2044-8309.1997.tb01123.x

[B45] Smith E. R. Fazio R. H. Cejka M. A. (1996). Accessible attitudes influence categorization of multiply categorizable objects. J. Pers. Soc. Psychol. 71, 888–898. 10.1037/0022-3514.71.5.888 8939039

[B46] Smith E. R. Stewart T. L. Buttram R. T. (1992). Inferring a trait from a behavior has long-term, highly specific effects. J. Pers. Soc. Psychol. 62, 753–759. 10.1037/0022-3514.62.5.753

[B47] Song J. Zuo B. Wen F. Tan X. Zhao M. (in press). The effect of crossed categorization on stereotype-wealth × age group. Psychol. Explor.

[B48] Turner J. C. Hogg M. A. Oakes P. J. Reicher S. D. Wetherell M. S. (1987). Rediscovering the Social Group: A Self-Categorization Theory. Oxford: Basil Blackwell.

[B49] Urada D. Stenstrom D. M. Miller N. (2007). Crossed categorization beyond the two-group model. J. Pers. Soc. Psychol. 92, 649–664. 10.1037/0022-3514.92.4.649 17469950

[B50] Van Rijswijk W. Ellemers N. (2002). Context effects on the application of stereotype content to multiple categorizable targets. Pers. Soc. Psychol. Bull. 28, 90–101. 10.1177/0146167202281008

