# Supplementary material for: Functional Significance of Conflicting Age and Wealth Cross-Categorization: The Dominant Role of Categories That Violate Stereotypical Expectations
Source: Front Psychol. 2016 Oct 21;7:1624. doi: 10.3389/fpsyg.2016.01624 (PMC5073204; doi:10.3389/fpsyg.2016.01624)
Supplement: Supplementary file 1 [file Presentation1.PDF]

Appendix A

**Pilot study**

Regard to the un-conflicting stereotype group, Chi-square tests were conducted to compare the number of people choosing 0, 1, 2 or 3 times the high competence/warmth box for each target with the expected value of 45/4=11.25. The frequencies with which participants placed all three old-poor targets into the low competence box ($n$=38) were significantly higher than the expected value ($\chi^2$= 85.22, df=3, $p$ <0.001), and there is not significant difference between the observed value and the expected value in the warmth evaluation of the old-poor target ($\chi^2$=2.56, df=3, $p$ >0.05). The frequencies with which participants placed all three young-rich targets into the high competence box ($n$=30) were significantly higher than the expected value ($\chi^2$=42.38, df=3, $p$ <0.001), but there is not significant difference between the observed value and the expected value in the warmth evaluation of the young-rich target ($\chi^2$=5.58, df=3, $p$ >0.05).

Moreover, the results showed that there is not significant difference between the observed value and the expected value in the warmth evaluation of the young-poor target ($\chi^2$=5.58, df=3, $p$ >0.05). The frequencies with which participants placed all three old-rich targets into the high competence box ($n$=35) were significantly higher than the expected value ($\chi^2$=67.98, df=3, $p$ <0.001).

Appendix B. Correlations among competence ratings for old, rich, and old-rich, and the warmth ratings for young, poor, and young-poor targets

| | 1.Old competence | 2.Rich competence | 3.Old-rich competence | 4.Young warmth | 5.Poor warmth | 6.Young-poor warmth |
|---|---|---|---|---|---|---|
| 1 | - | | | | | |
| 2 | 0.07 | - | | | | |
| 3 | 0.29** | 0.46** | - | | | |
| 4 | 0.19* | 0.38** | 0.08 | - | | |
| 5 | 0.18 | 0.36** | 0.11 | 0.38** | - | |
| 6 | 0.11 | 0.23 | -0.06 | 0.36** | 0.49** | - |
| *M* | 8.71 | 11.62 | 11.04 | 10.21 | 10.23 | 9.96 |
| *SD* | 2.07 | 2.15 | 2.34 | 2.12 | 2.56 | 2.46 |

Note: $N$=104 $^{*}p<0.05$, $^{**}p<0.01$, $^{***}p<0.001$, scale range: 3-15.

Appendix C. Hierarchical Linear Models of simple-category evaluations in relation to crossed-category evaluations (N=104)

| Competence evaluation | | | Warmth evaluation | | |
|---|---|---|---|---|---|
| | $\beta$ | $t$ | | $\beta$ | $t$ |
| Age | 0.04 | 0.41 | Age | 0.15 | 1.57 |
| Gender | -0.05 | -0.48 | Gender | -0.16 | -1.81 |
| Wealth | 0.02 | 0.24 | Wealth | -0.15 | -1.79 |
| Hukou | -0.12 | -0.13 | Hukou | 0.08 | 0.89 |
| Old | 0.26 | $2.98^{**}$ | Young | 0.19 | $2.05^{*}$ |
| Rich | 0.45 | $5.03^{***}$ | Poor | 0.41 | $4.54^{***}$ |
| | $R^2$=0.29 | | | $R^2$=0.32 | |

Note: Hukou is a household registration system in China, and it includes two types: rural and city, 1 = city, 2 = rural. For Gender, 1=male, 2=female; $^{*}p<0.05$, $^{**}p<0.01$, $^{***}p<0.001$

Appendix D. the categories of attributions in two warmth and two scenarios

| | **High warmth scenario** | **Low warmth scenario** |
|---|---|---|
| Categories of attributions in the warmth scenario | The target helps the stranger because of external benefits (e.g., personal reputation and image, tips). | Her/his low warmth behavior may be because of personal internal attributes (e.g., the target just does not want to help the stranger as she/he is very indifferent and ruthless). |
| | The target helps the stranger because of psychological benefits (e.g., atonement or self-redemption). | The target doesn't want to help the stranger because of situational factors (e.g., she/he doesn't want to help the stranger, as she/he is too busy to care about others). |
| | The target helps the stranger because of situational factors (e.g., not busy, has a good temper at that time). | The target doesn't help the stranger because she/he is afraid that the stranger is a liar or a thief. |
| | The target helps the stranger because she/he sympathizes with the disabled, or she/he could understand the feelings of the stranger as she/he always needs others' help. | The target may want to help the stranger, but she/he can't because of situational factors (e.g., she/he may want to help, but she/he is busy at that time). |
| | The target helps the stranger because of personal internal attributes (e.g., she/he is a very kind and warm person). | Her/his low warmth behavior may be because of external factors (e.g., the target is willing to help the stranger, but she/he doesn't know the way to get there). |
| | **High competence scenario** | **Low competence scenario** |
| Categories of attributions in the competence scenario | Her/his success may be because of external factors (e.g., good luck, she/he is the boss's relative). | Her/his failure may be because of her/his personal internal attributes (e.g., low competence). |
| | | Her/his failure may be because her/his view, opinion, and ideas were limited because of living in a rural area. |
| | Her/his success may be because of personal attributes that are not related to competence (e.g., high warmth, good relationship with colleagues). | Her/his failure may be because of her/his temperament (e.g., self-assertiveness, conceit, or low self-esteem). |
| | Her/his success may be because of the efforts she/he made (e.g., works hard, more exercise, more experience). | She/he may have high competence, and her/his failure may be just because of less experience with job interviews (e.g., poor preparation, wears shabby clothes, not highly educated) |
| | Her/his success may be because of personal internal attributes (e.g., has high competence and high creativity). | Her/his failure may be because of external factors (e.g., she/he has high competence, but the rival is the boss's relative). |
