# Supplementary material for: Functional Significance of Conflicting Age and Wealth Cross-Categorization: The Dominant Role of Categories That Violate Stereotypical Expectations
Source: Front Psychol. 2016 Oct 21;7:1624. doi: 10.3389/fpsyg.2016.01624 (PMC5073204; doi:10.3389/fpsyg.2016.01624)

Supplementary materials

The Categorization Task

The Categorization Task: The task consisted of 8 “identity cards”. Each identity cards present e very simple head-and-shoulders photos silhouette in black-and-white colour in the left of the card(Dommelen, Schmid, Hewstone, Gonsalkorale, & Brewer, 2015), and present the identity information in text in the right of the cards.

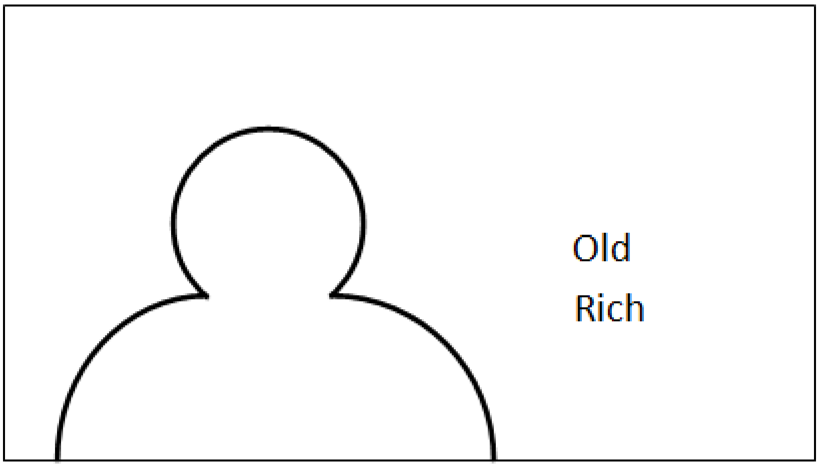

Supplement: Supplementary file 2 [file Image1.PDF]
